# Plastome evolution and phylogenomic insights from representative taxa across all nine subfamilies of Malvaceae

**DOI:** 10.1186/s12870-026-09562-y

**Published:** 2026-07-18

**Authors:** Wen Deng, Hui-Long Li, Wei Gu, Kai-Lun An, Li-Bo Zhao, Xiao-Gang Fu, Ding-Jie Wang, Si-Yun Chen, Cheng Liu, Xue-Li Zhao, Ting-Shuang Yi, Rong Zhang

**Affiliations:** 1 College of Forestry, Southwest Forestry University, Kunming, 650224 China; 2 Germplasm Bank of Wild Species & Yunnan Key Laboratory of Crop Wild Relatives Omics, Kunming Institute of Botany, Chinese Academy of Sciences, Kunming, 650201 China

**Keywords:** DNA markers, Malvaceae, Phylogenomics, Plastomes, Positive selection, Structural variation

## Abstract

Malvaceae Juss. comprises nine subfamilies and approximately 4,225 species, many of which are ecologically significant and several of which are cultivated for their high economic value. However, the backbone phylogeny of the family remains poorly resolved, and comprehensive structural variations across the entire family’s plastomes are not comprehensively investigated. We newly sequenced 45 Malvaceae samples and integrated them with 85 plastomes from GenBank for phylogenetic analyses. After excluding two outgroups and four plastomes containing gaps, 124 Malvaceae plastomes were used for comparative structural analyses. The newly assembled plastomes exhibit a typical quadripartite structure, with length ranging from 158,346 to 163,741 bp and encoding 129–132 genes. We identified five distinct types of boundaries between inverted repeat regions (IRs) and single-copy (SC) regions. Furthermore, six coding genes (e.g., *matK*, *ndhF*) and 14 non-coding regions (e.g., *trnH-psbA*) were identified as highly variable, providing potential DNA markers for species delimitation. Analyses of simple sequence repeats (SSRs) and long repeats revealed unique repeat patterns in *Durio* Adans., while codon usage bias analysis revealed a strong A/T preference across this family. Selection pressure analysis detected positive selection (*dN/dS* > 1) in the *rpl23* gene, suggesting its potential role in adaptive evolution. Phylogenomic analyses reconstructed a highly supported backbone topology, resolving Malvaceae into two major clades: Byttneriina and Malvadendrina. Within Malvadendrina, Helicteroideae was the earliest-diverging lineage, and Tilioideae was strongly supported as sister to Dombeyoideae. Additionally, *Hibiscus* L. and *Sida* L. were confirmed to be non-monophyletic. This study clarifies the major phylogenetic relationships within Malvaceae using a densely sampled plastome dataset and provides novel insights into the structural features and evolution of Malvaceae plastomes.

**Supplementary Information:**

The online version contains supplementary material available at https://doi.org/10.1186/s12870-026-09562-y.

## Introduction

Malvaceae Juss. is globally distributed across tropical and temperate zones in both hemispheres, with many are ecologically and economically important. The family currently comprises approximately 244 genera and over 4,225 species, with these numbers continually increasing as new species are described [1, 2]. Its species exhibit remarkable morphological diversity, spanning herbaceous plants to large woody trees [3]. Additionally, Malvaceae possesses significant economic value in global agriculture, medicine, and horticulture. This family provides essential natural fibers and food resources; for example, *Gossypium* L. (cotton) and *Ceiba* Mill. are crucial for the global textile industry [2, 4], while *Theobroma cacao* L. (cacao), *Abelmoschus esculentus* (L.) Moench. (okra), and *Durio zibethinus* L. (durian) are key food crops known for their unique nutritional profiles [5, 6]. The family is also a rich source of bioactive compounds, as seen in *Cola acuminata* (P.Beauv.) Schott & Endl., which produces diverse secondary metabolites with significant antioxidant, antibacterial, and anti-inflammatory properties, supporting its long-standing use in traditional medicine [7]. Furthermore, many species, such as those in *Hibiscus* L., are highly valued ornamentals [1, 8].

The taxonomic history of Malvaceae has undergone significant revisions. Traditional classifications divided the core Malvales into four families (Malvaceae, Sterculiaceae, Bombacaceae, and Tiliaceae), but phylogenetic studies revealed them to be polyphyletic or paraphyletic. Consequently, the APG system merged them into a broadly defined Malvaceae *s.l.*, now divided into two primary clades—the Byttneriina clade (including Byttnerioideae and Grewioideae) and the Malvadendrina clade (comprising Malvoideae, Bombacoideae, Brownlowioideae, Dombeyoideae, Helicteroideae, Sterculioideae, and Tilioideae) [9–11]. While this two-clade framework is well-supported, relationships among subfamilies within the Malvadendrina clade remain contentious [12, 13]. For example, the placements of Sterculioideae, Dombeyoideae, Tilioideae, and Helicteroideae have been inconsistently resolved across different studies [14, 15]. Some studies have supported a clade comprising Brownlowioideae, Dombeyoideae, and Tilioideae as sister to the Malvatheca clade with moderate bootstrap support (BS = 72%) [16], while others have suggested a sister relationship between Dombeyoideae and Brownlowioideae, albeit with weak support (BS = 38%, Posterior Probability = 0.72) [13]. Notably, recent phylogenomic analyses have revealed discordance between nuclear and plastome datasets regarding the first-diverging lineage of Malvadendrina, with nuclear genes supporting Dombeyoideae and plastome data strongly supporting Helicteroideae (represented by *Durio L.*) [15]. A highly resolved phylogeny of Malvaceae was reconstructed using 309 nuclear genes [17] revealed Helicteroideae as the first-diverging clade within Malvadendrina, and resolved relationships among Sterculioideae, Brownlowioideae, Dombeyoideae, and Tilioideae that were incongruent with those reconstructed from plastome data [8, 14]. Plastomic studies have supported that Byttnerioideae and Grewioideae form a clade, while the remaining seven subfamilies constitute another [15, 18, 19]. These phylogenetic uncertainties primarily stem from insufficient taxon sampling, a limited number of informative sites, and complex evolutionary histories.

The plastome of angiosperms typically exhibits slower substitution rates and a highly conserved genomic structures compared to nuclear genomes [20]. They are generally circular and exhibit a quadripartite structure, comprising one large single-copy region (LSC), one small single-copy region (SSC), and two inverted repeat regions (IRs). They typically range from 120 to 160 kb in size and encode 110 to 130 genes [21]. Their usual maternal inheritance and general lack of recombination, coupled with a moderate evolutionary rate, offer unique advantages for phylogenetic reconstruction [22, 23]. Although highly conserved, plastomes can exhibit variations in size, structure, and gene evolution [24, 25], providing insights into organellar evolution, biodiversity, and adaptation [26–29]. Therefore, integrating plastome data with expanded taxon sampling is crucial for clarifying phylogenetic relationships within Malvaceae.

In this study, we used 130 plastomes from 127 Malvaceae species (128 samples) across all nine subfamilies and two outgroups including 45 newly sequenced and 85 previously published ones for phylogenetic analyses. For structural analyses, we excluded four plastomes containing "N" gaps and two outgroup plastomes, resulting in a final dataset of 124 plastomes. Our objectives are threefold: (1) to characterize plastome structural features across Malvaceae; (2) to identify hypervariable regions as potential molecular markers for phylogenetics and species identification; (3) to reconstruct a robust phylogenetic framework for all nine subfamilies using this large-scale plastome dataset. This work will improve our understanding of plastome evolution in Malvaceae and provide valuable genetic resources for future research.

## Materials and methods

### Sampling and sequencing

We analyzed a total of 130 samples, including 128 Malvaceae samples (representing 127 species) and two outgroup species, *Aquilaria crassna* Pierre ex Lecomte. (Thymelaeaceae) and *Dipterocarpus turbinatus* C.F.Gaertn. (Dipterocarpaceae). The dataset comprised 85 previously assembled plastomes from GenBank and 45 newly sequenced plastomes (Table S1 and Table S2). For plastome structural variation analysis, we used 124 plastomes, having excluded four plastomes (*Durio kutejensis (Hassk.) Becc.*, *Durio testudinarius Becc.*, and *Pterospermum kingtungense* C.Y.Wu ex H.H.Hsue. from GenBank, and *Kydia calycina* Roxb. from newly sequenced data) due to the presence of ambiguous bases ("N"). For the 45 newly sequenced samples, total DNA was extracted from fresh, silica-dried and herbarium specimens' leaves using the CTAB method [30]. All 45 newly sequenced plastomes were successfully assembled and used for phylogenetic analyses; however, the plastome of *Kydia calycina* contained Ns and was therefore excluded from the structural variation analysis. The total DNA was used to construct paired-end libraries following Illumina’s standard protocols. Sequencing was performed on the Illumina Hiseq XTen platform at the Molecular Biology Experiment Platform, the Germplasm Bank of Wild Species, Kunming Institute of Botany, Chinese Academy of Sciences.

For the newly sequenced samples in this study, molecular materials were obtained through institutional collaboration from existing herbarium collections of the Herbarium, Kunming Institute of Botany, Chinese Academy of Sciences (KUN), following standard sampling procedures. Detailed information for each sampled taxon, including species name, laboratory code, collector number, voucher specimen number, voucher repository, source locality, identifier, sequencing platform, sequencing type, and the accession number in the China National GeneBank Sequence Archive (CNSA), is provided in Table S2. Formal identification of the sampled materials was based on the identification information indicated on the original specimen labels. Voucher specimens corresponding to the sampled materials are deposited in KUN.

### Plastome assembly and annotation

The 45 newly sequenced plastomes were assembled from raw sequencing data using GetOrganelle v1.6.2d [24]. Initial annotation was performed with PGA2.0 [31, 32], and annotation quality was assessed using the check_annotations.py script (https://github.com/Kinggerm/GetOrganelle). To verify annotations, these plastomes were also annotated with the web-based tool GeSeq [33]. Final manual curation of the annotations was carried out in Geneious v9.1.8 [34]. Circular plastome maps were generated using the online tool OGDRAW [35]. Genome collinearity analysis was performed with the ProgressiveMauve plugin in Geneious v9.1.8 [34, 36].

### Border comparison and identification of divergence hotspot regions

The IR boundaries of selected 124 Malvaceae plastomes were visualized using the online program CPJSDraw [37], illustrating the LSC, SSC, and IR regions. For the comparative genomic analysis, we selected representative species from each of the 38 genera present in the 124 plastomes. Using *Abelmoschus crinitus Wall. (S07890)* as the reference, the analysis was performed with mVISTA [38] under the shuffle-LAGAN alignment mode. Nucleotide diversity (Pi) of protein-coding genes (PCGs) and intergenic spacers (IGSs) was calculated with the “Pi” plugin implemented in CPStools v1.2 [39].

### Codon usage analysis and repeat sequences identification

Codon usage statistics for protein-coding genes (PCGs) were generated using CPStools [39]. Simple sequence repeats (SSRs) were detected with the integrated “SSRs” plugin of CPStools [39], with threshold values set at 10, 5, 4, 3, 3, and 3 for mono-, di-, tri-, tetra-, penta-, and hexanucleotide repeats, respectively. Four types of long repeat sequences—forward (F), reverse (R), complement (C), and palindromic (P)—were identified using REPuter [40, 41]. Parameters were set as follows: a minimal repeat size of 30 bp, a Hamming distance of 3, and a maximum of 5,000 computed repeats. All 124 complete plastomes of Malvaceae were included in these analyses.

### Phylogenetic analysis

Phylogenetic trees were reconstructed using the maximum likelihood (ML) and Bayesian inference (BI) methods based on two datasets: the complete plastome and the plastomic coding sequences. The analysis included 128 samples representing 127 species of Malvaceae and two outgroup species. Protein coding genes were extracted from the plastomes of all samples using a Python script, "get_annotated_regions_from_gb.py" (https://github.com/Kinggerm/PersonalUtilities). All gene sequences were aligned with the MAFFT plugin in PhyloSuite v1.2.3 [42], using default parameters. The aligned sequences were then concatenated into separate supermatrices utilizing the ‘concatenate’ function within PhyloSuite. Finally, maximum likelihood trees for each matrix were constructed with RAxML v8.2.12 [43], employing the GTRGAMMA substitution model and performing 1000 bootstrap replicates to assess branch support. In addition, BI analyses were performed for both datasets using MrBayes v3.2.7 under the GTR + G model [44]. For each analysis, two independent MCMC runs were conducted, each with four chains, for 10,000,000 generations, sampling every 1000 generations. The first 25% of samples were discarded as burn-in, and the remaining samples were used to generate a 50% majority-rule consensus tree, with posterior probabilities (PP) calculated for each node.

### Selection pressure analyses

For selection pressure analyses, we used the same set of 78 protein-coding genes as that used to construct the CDS phylogenetic tree. These genes were present in all 124 plastomes included in the analysis, as confirmed by sequence alignments and a gene presence/absence matrix. Genes absent from one or more taxa, showing poor alignment quality, or unsuitable for reliable codon-based comparison were excluded. The retained genes were further checked for sequence integrity, including reading-frame continuity, absence of internal premature stop codons, and no obvious truncations or pseudogenization features. The full list of the 78 genes and their *dN/dS* values is provided in Table S8. For *rpl23* in particular, we additionally confirmed the absence of frameshifts, internal stop codons, truncations, and annotation errors in all 124 plastomes.

Protein-coding genes were aligned using the codon alignment option of the MAFFT plugin in PhyloSuite [42] with default parameters. The aligned sequences were then converted to the "PML" format using the "Convert Sequence Format" tool within the same software. The nonsynonymous/synonymous substitution rate ratio (*dN/dS* ratio) was calculated using the CodeML program in PAML v4.10.7 [45], with the ML tree of PCGs-Con and the PML-formatted aligned sequences as inputs. We employed the branch model for selection pressure analysis. Specifically, two models were tested: the one-ratio model (assuming a uniform ω across all branches; parameters: model = 0, NSites = 0) and the two-ratio model (assigning distinct ω values to foreground versus background branches; parameters: model = 2, NSites = 0). In the latter, designated clades were set as foreground branches, while all other branches were treated as background. Differences in selection pressure among branches for each PCG were assessed via a likelihood ratio test (LRT) [46]. Foreground branches were designated using EasyCodeML v1.0 [47], and all configurations followed the PAML manual specifications.

## Results

### Plastome genome size and characteristics

**Fig. 1:**
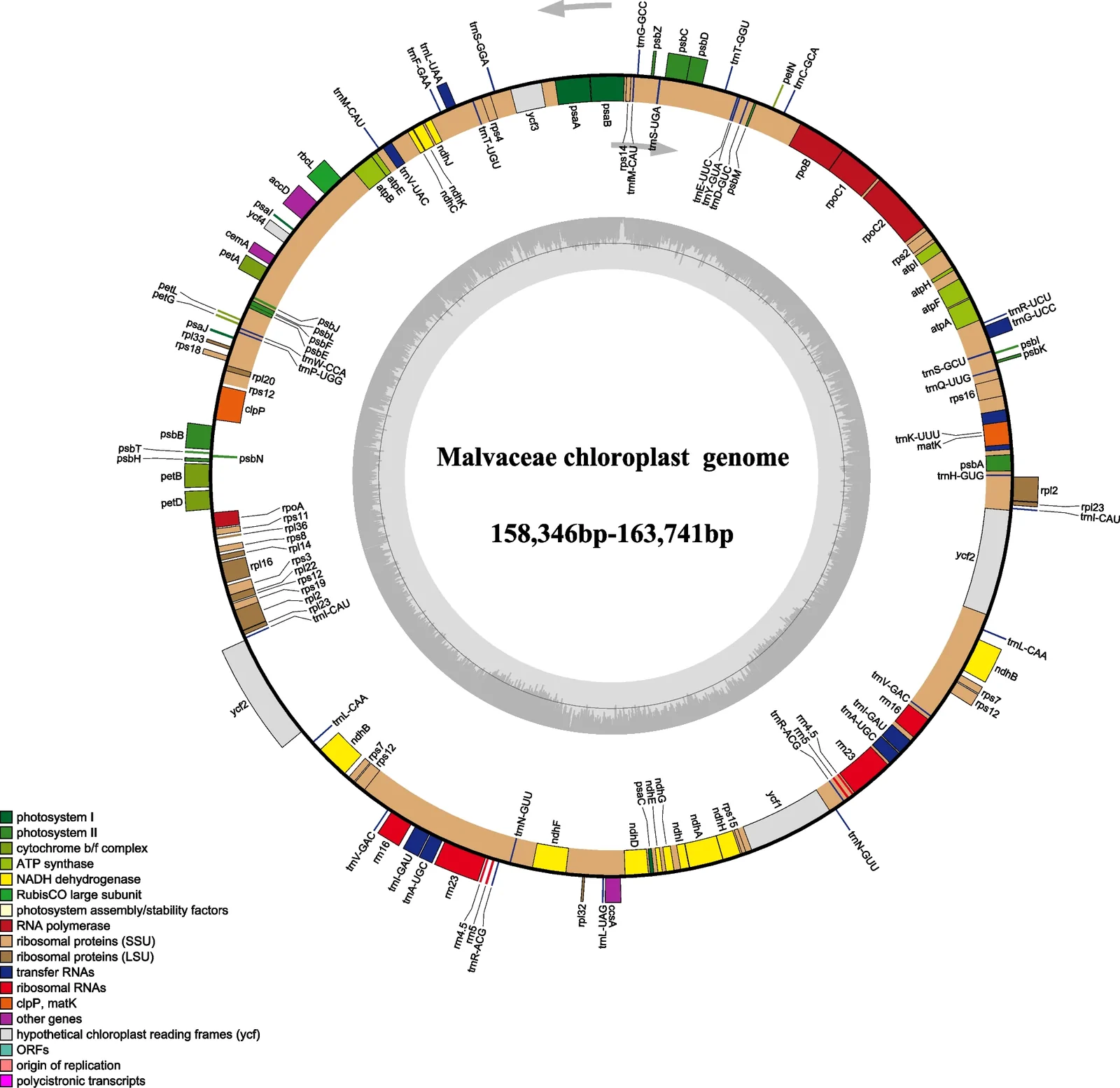
The plastome map of Malvaceae. Genes inside the circle are transcribed clockwise, while genes outside the circle are transcribed counterclockwise. Genes belonging to different functional groups are color-coded. The darker and lighter gray areas in the inner circle corresponds to the GC and AT content, respectively

The 44 newly sequenced gap-free plastomes of Malvaceae used for comparative structural analyses were derived from the original 45 newly sequenced plastomes after excluding one species, *Kydia calycina* (162691 bp), because its sequence contained ambiguous bases (“N”). All exhibited the typical quadripartite structure (LSC, SSC, and a pair of IRs) (e.g. Fig. 1). The total length of these plastomes ranged from 158,346 to 163,741 bp. The LSC, SSC, and IR regions spanned 87,051–91,213 bp, 18,886–21,142 bp, and 25,095–28,171 bp, respectively. The overall GC content varied between 36.48% and 37.31% (Table S3). All 124 plastomes analyzed showed strong collinearity and had identical gene content and arrangement (Fig. S1). These plastomes contained 129–132 annotated genes, including 84–87 protein-coding genes (PCGs), 37 tRNA genes, and 8 rRNA genes (Fig. 1; Tables S3 and S4). Typically, 12 PCGs were located in the IRs; however, this number increased to 18 in *Abelmoschus*. Of the tRNA genes, 14 were in the IRs; all 8 rRNA genes were located within IRs.

### Phylogenetic inferences

**Fig. 2:**
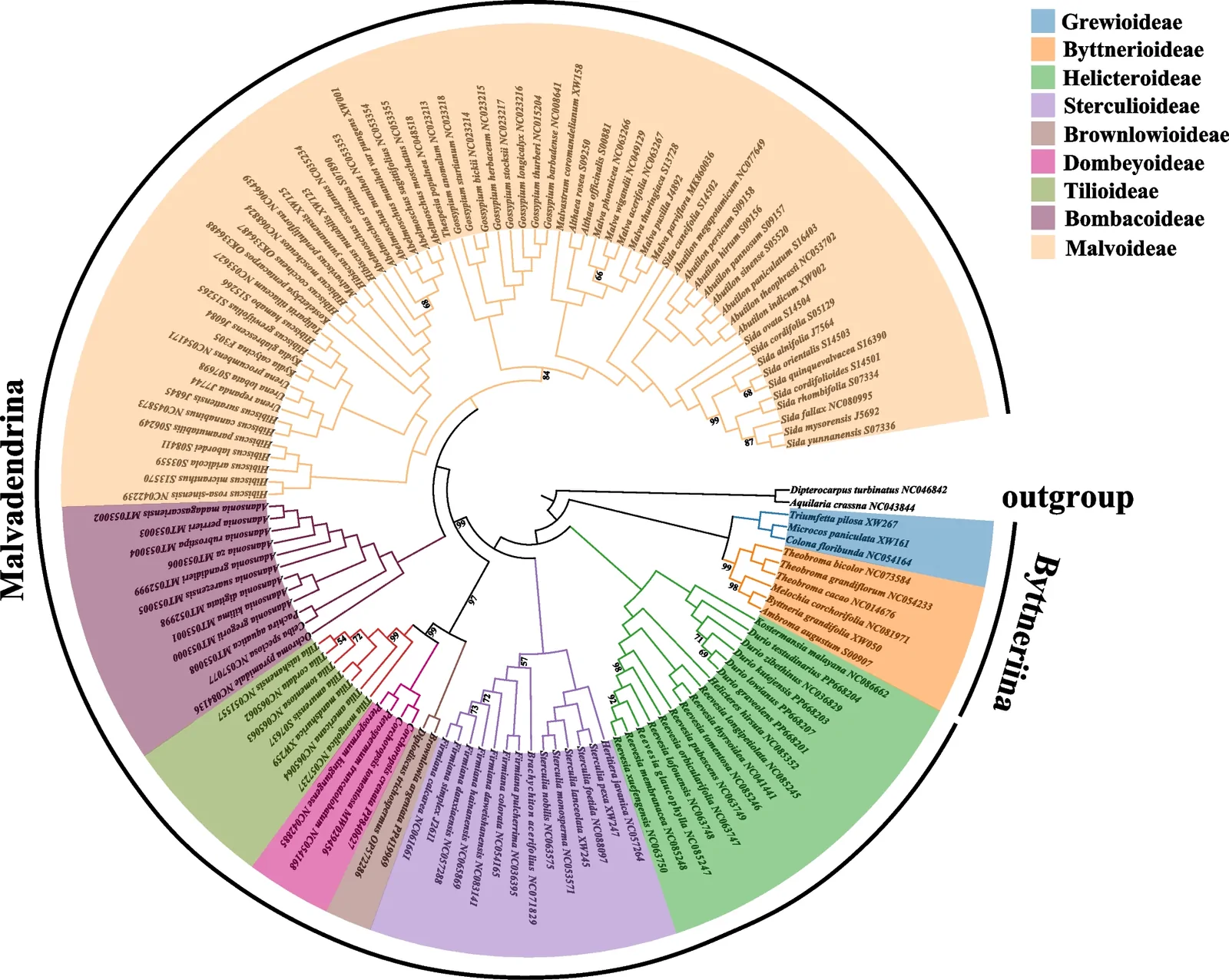
Maximum likelihood phylogenetic tree of Malvaceae inferred from complete plastome sequences of 130 taxa, including 128 Malvaceae samples representing 127 species and two outgroups. Bootstrap support values are shown at nodes; nodes without indicated values received 100% support

Phylogenetic trees reconstructed from complete plastome and coding sequence (CDS/PCG) matrices were largely congruent, with most nodes having strong support (BS = 100% and PP = 1.00) (Fig. 2; Fig S2-S4). However, a topological discrepancy was observed in the placement of *Theobroma* between the two datasets. For each dataset, the ML and BI trees were completely identical in topology. In the complete plastome tree, *Theobroma* was recovered within Byttnerioideae with strong support (BS = 99% and PP = 1.00), whereas in the CDS tree, it was alternatively placed within Grewioideae with moderate support (BS = 88% and PP = 1.00). The plastome tree, which had the highest nodal support, was used for subsequent analyses. The results confirmed that Byttnerioideae and Grewioideae together form the Byttneriina clade, while the remaining seven subfamilies constitute the Malvadendrina clade. Within Malvadendrina, Helicteroideae was resolved as the earliest-diverging lineage (BS = 100% and PP = 1.00), followed by Sterculioideae (BS = 100% and PP = 1.00). Brownlowioideae was sister (BS = 97% and PP = 1.00) to a strongly supported (BS = 99% and PP = 1.00) clade comprising Tilioideae and Dombeyoideae. This phylogenetic topology is consistent with previous studies [8, 48]. Furthermore, both *Sida* and *Hibiscus* (Malvoideae) were found to be non-monophyletic.

### Comparisons of IR boundaries

**Fig. 3:**
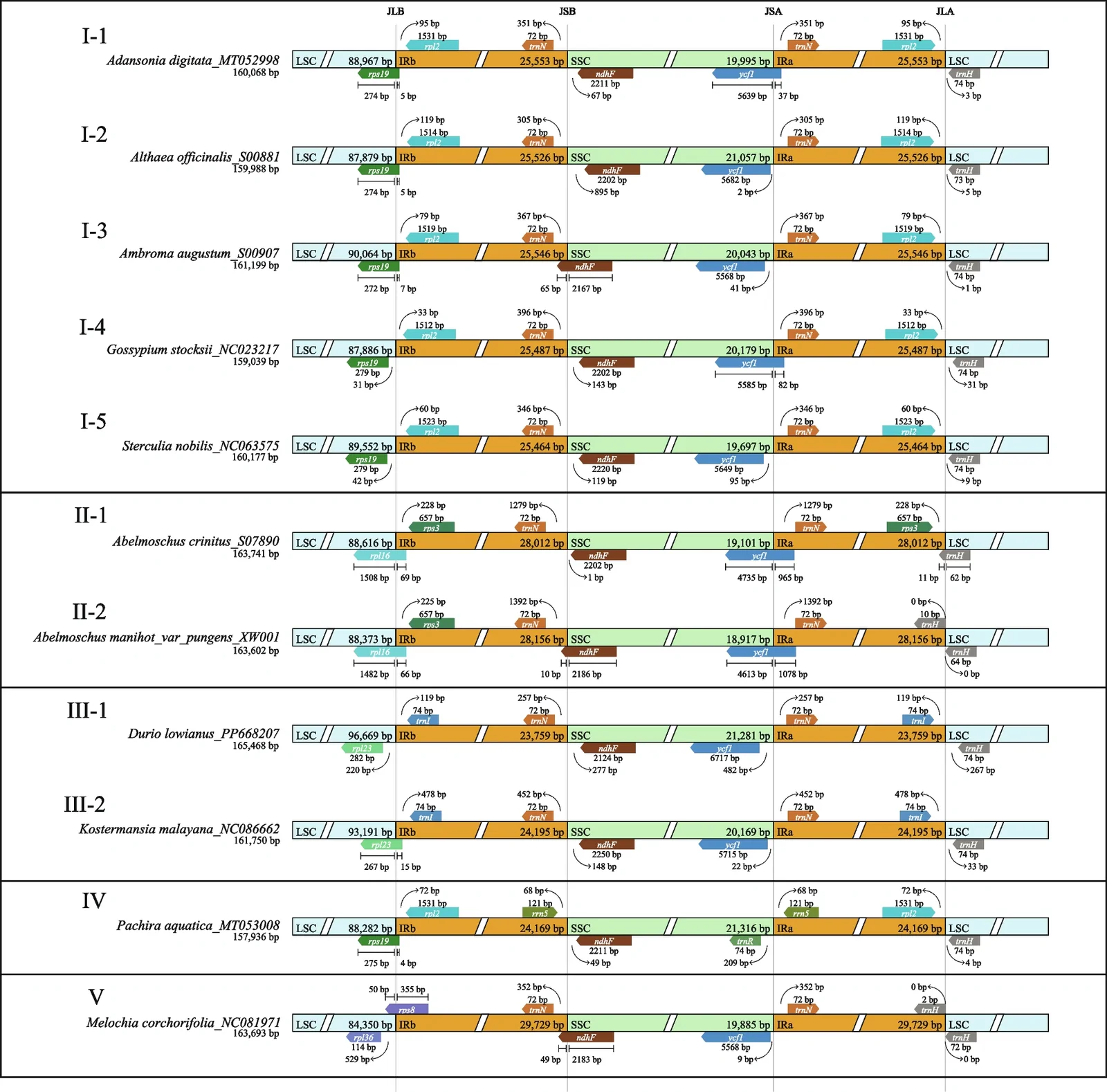
Five types and 11 subtypes of inverted repeat/single-copy boundaries identified in 124 Malvaceae plastomes. This figure is not to scale. The inverted repeat (IR), large single-copy (LSC), and small single-copy (SSC) regions are shown for each boundary type

Analysis of 124 Malvaceae plastomes revealed five distinct types of inverted repeat/single-copy (IR/SC) boundaries, comprising 11 specific subtypes (Fig. 3, Fig. S5). Type I, the most common type, present in 112 plastomes, was characterized by the absence of major IR expansion/contraction. The boundaries were defined as follows: the LSC/IRb boundary (JLB) was located within the *rps19* gene or between *rps19* and *rpl2*; the IRb/SSC boundary (JSB) was located within the *ndhF* gene or between *trnN* and *ndhF*; the SSC/IRa boundary (JSA) occurred within the *ycf1* gene or between *ycf1* and *trnN*; and the IRa/LSC boundary (JLA) was situated between *rpl2* and *trnH*(GUG). Type I was further subdivided into five subtypes based on boundary positions (Fig. 3). Subtype I-1 was found in 76 plastomes, with JLB within *rps19*, JSB between *trnN* and *ndhF*, JSA within *ycf1*, and JLA between *rpl2* and *trnH*(GUG). Subtype I-2 was present in 17 plastomes, with JLB within *rps19*, JSB between *trnN* and *ndhF*, JSA between *ycf1* and *trnN*, and JLA between *rpl2* and *trnH*(GUG). Subtype I-3 occurred in five plastomes, with JLB within *rps19*, JSB within *ndhF*, JSA between *ycf1* and *trnN*, and JLA between *rpl2* and *trnH*(GUG). Subtype I-4 was identified in 12 plastomes, with JLB between *rps19* and *rpl2*, JSB between *trnN* and *ndhF*, JSA within *ycf1*, and JLA between *rpl2* and *trnH*(GUG). Subtype I-5 was found in two plastomes, with JLB between *rps19* and *rpl2*, JSB between *trnN* and *ndhF*, JSA between *ycf1* and *trnN*, and JLA between *rpl2* and *trnH*(GUG).

Types II-V exhibited IR boundary variations in other Malvaceae plastomes. Type II showed IR expansion to varying degrees towards both the SSC and LSC regions. Subtype II-1 was exemplified by the plastome of *A. crinitus*, with *rpl16* spanning JLB (1,508 bp in LSC and 69 bp in IRb), while *ycf1* spanning JSA (4,735 bp in SSC and 695 bp in IRa); this subtype was found in two plastomes. Subtype II-2 was exemplified by the plastome of *Abelmoschus manihot var. pungens (Roxb.) Hochr.*, with *rpl16* spanning JLB (1,482 bp in LSC and 66 bp in IRb), *ndhF* spanning JSB (2,186 bp in SSC and 10 bp in IRb), and *ycf1* spanning JSA (4,613 bp in SSC and 1,087 bp in IRa), this subtype was present in four plastomes. Type III featured IR contraction, with *ndhF* and *ycf1* fully located within SSC; this type was found only in four *Durio* and *Kostermansia* Soegeng. species. Type IV was unique to the plastome of *Pachira aquatica Aubl.*, which also showed IR contraction with *ndhF* and *trnR* genes completely within SSC. Type V was solely identified in the plastome of *Melochia corchorifolia Wall.*, which displayed IR expansion into both the LSC and SSC regions; *rps8* spanning JLB with 50 bp in LSC and 355 bp in IRb, *ndhF* spanning JSA (2,183 bp in SSC and 49 bp in IRb), and *trnH*(GUG) spanning JLA (2 bp in SSC and 72 bp in LSC) (Fig. 3).

### Plastome sequence divergence

**Fig. 4:**
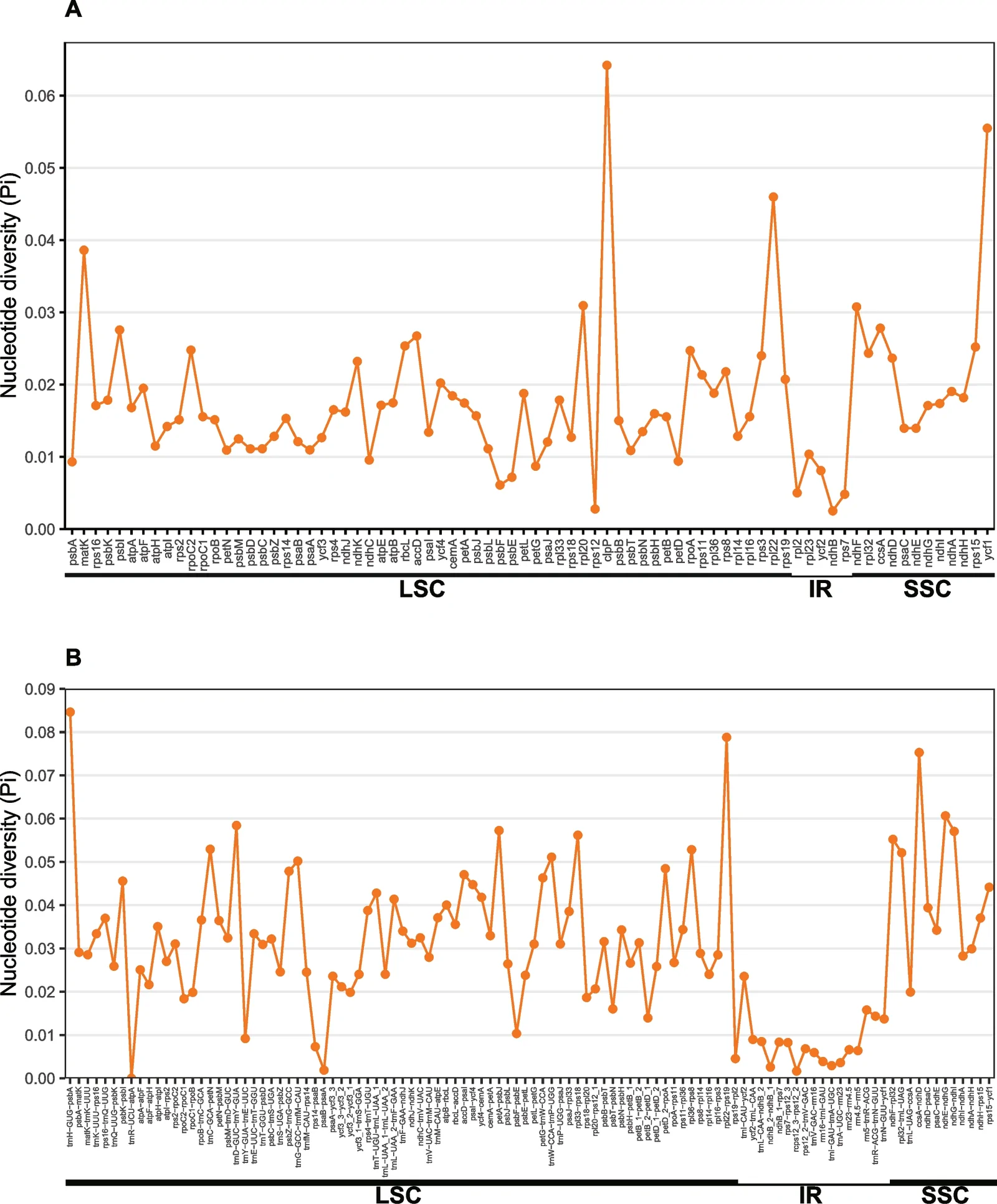
Nucleotide diversity (Pi) values of different regions of 124 Malvaceae plastomes. **A** Pi values of protein-coding regions; **B** Pi values of non-coding regions

Comparative analysis of 124 Malvaceae plastomes indicated that single-copy regions were more variable than the IR regions, and that non-coding regions were more variable than coding regions (Fig. 4). Comparative plastomic analysis of representative species from 38 genera was shown in Fig. S6. We identified a total of 14 highly variable non-coding regions and six variable coding regions (Fig. 4). Nucleotide diversity (Pi) analysis revealed that coding regions including *matK*, *rpl20*, *clpP*, *rpl22*, *ndhF*, and *ycf1* had Pi values > 0.03. Among non-coding regions, 14 regions including *trnH-GUG-psbA*, *trnC-GCA-petN*, *trnD-GUC-trnY-GUA*, *trnG-GCC-trnfM-CAU*, *petA-psbJ*, *trnW-CCA-trnP-UGG*, *rpl33-rps18*, *rpl36-rps8*, *rpl22-rps19*, *ndhF-rpl32*, *rpl32-trnL-UAG*, *ccsA-ndhD*, *ndhE-ndhG* and *ndhG-ndhI* showed Pi values > 0.05.

### Repeat sequences identification

**Fig. 5:**
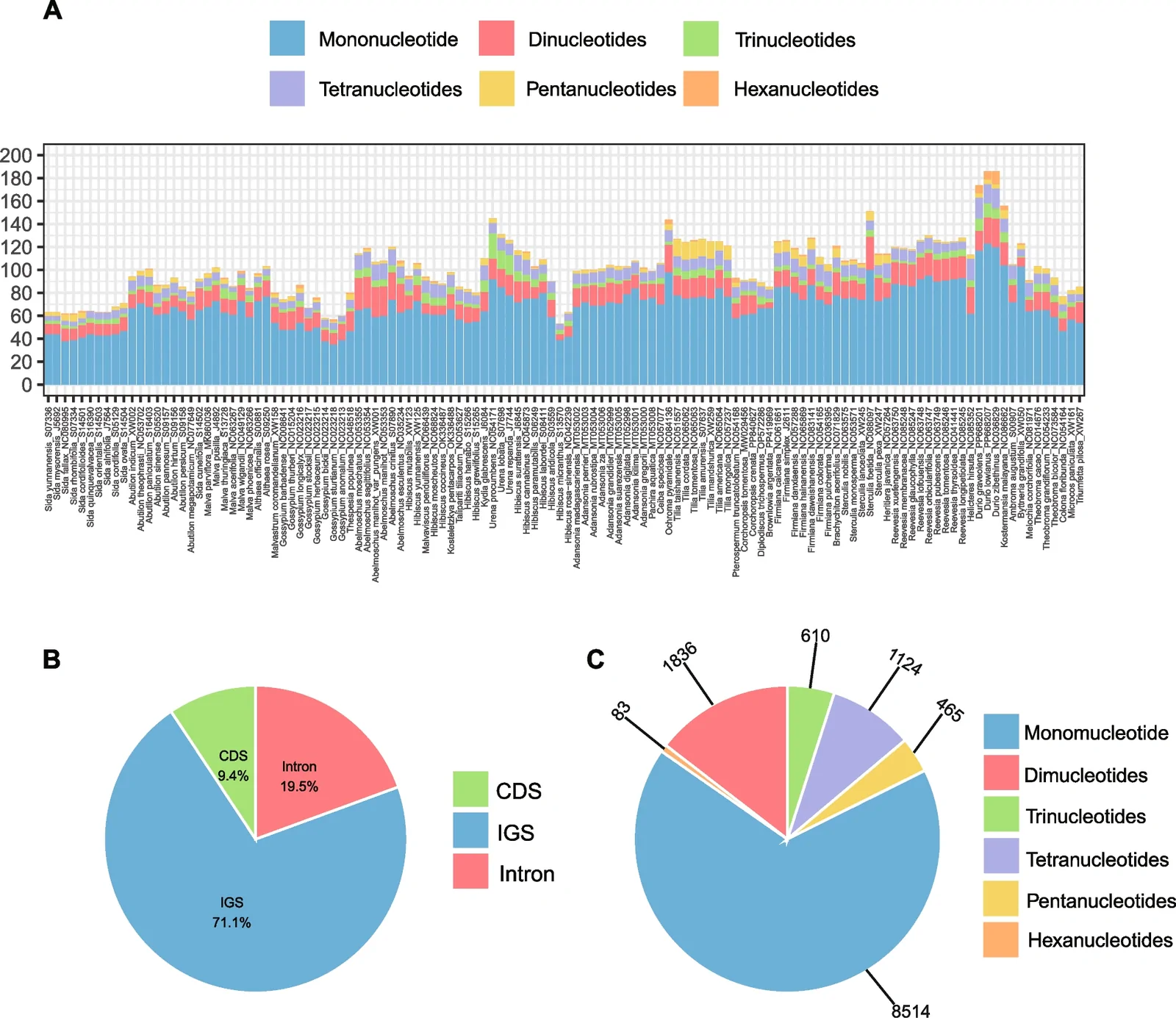
Analysis of simple sequence repeats (SSRs) in 124 plastomes of Malvaceae: **A** Statistics on the types and quantities of SSRs identified across different species; **B** Distribution patterns of SSRs in various regions of the plastomes; **C** Distribution of six types of SSRs identified in the analyzed plastomes

In the 124 Malvaceae plastomes, a total of 12,632 SSRs were identified. The number of SSRs per plastome varies from the lowest count of 53 in *Hibiscus micranthus L.f. to *the highest of 186 in *Durio zibethinus* (Fig. 5A and Table S5). Most SSRs were in the non-coding regions (Fig. 5B). Mononucleotide SSRs were most abundant (8,514), followed by dinucleotide (1,836) and tetranucleotide (1,124), trinucleotide (610), pentanucleotide (465), and hexanucleotide SSRs (83) (Fig. 5C).

**Fig. 6:**
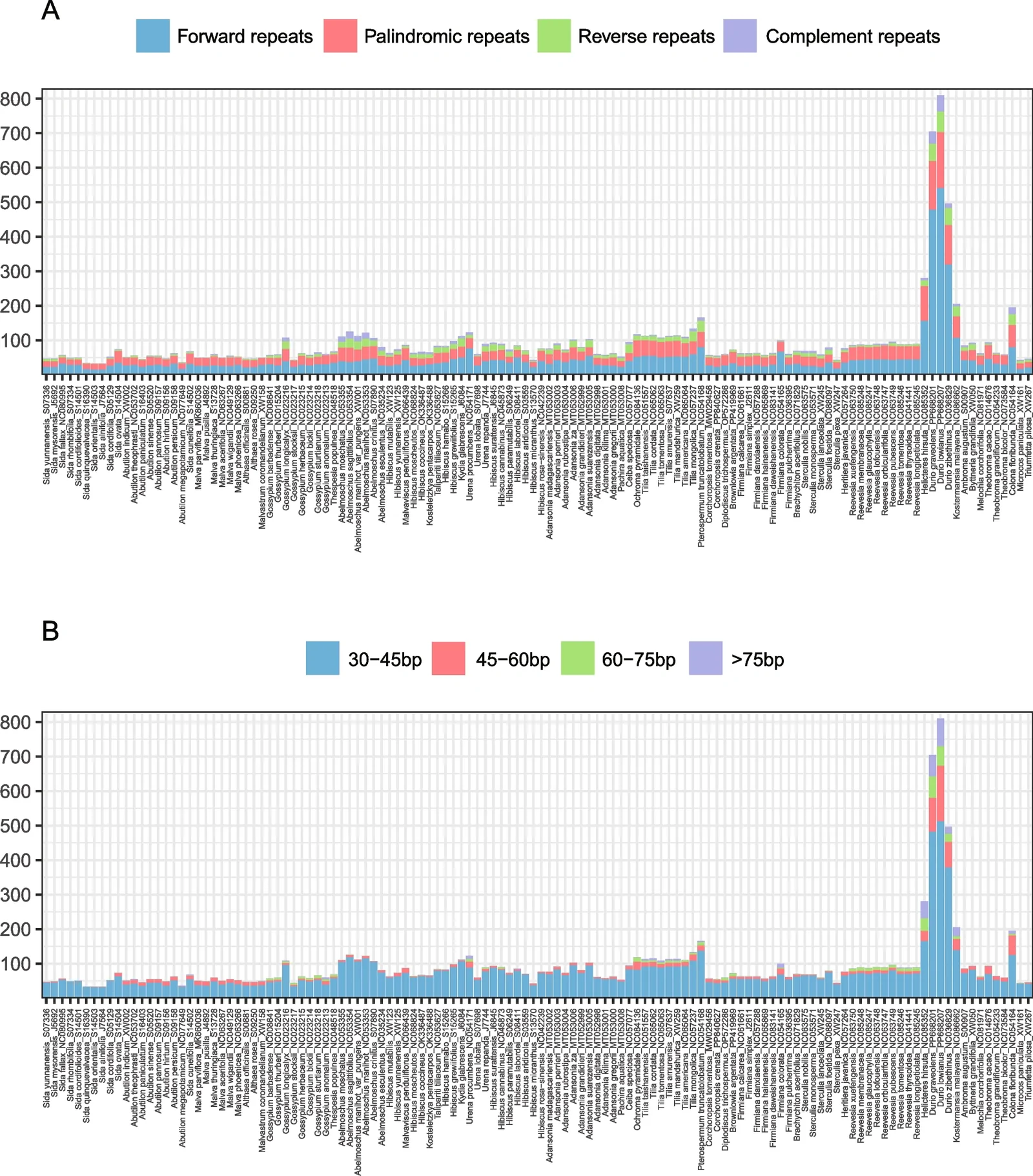
Analyses of long repeats in 124 Malvaceae plastomes.** A** Numbers of long repeats detected in the Malvaceae plastomes within each species; **B** Distribution of repeat counts by length category

A total of 11,416 long repeats were identified, comprising 5,841 forward 3,860 palindromic, 1,224 reverse, and 491 complementary repeats. The number of long repeats per plastome ranged from 33 in *Sida orientalis* Cav. and *Sida alnifolia* L. to 810 in *Durio lowianus Scort. ex King.*. Notably, *D. zibethinus*, *Durio graveolens Becc.*, and *D. lowianus* exhibited markedly high numbers of repeats: 496, 705, and 810, respectively. These were followed by *Pterospermum truncatolobatum Gagnep.*, *Colona floribunda (Kurz.) Craib.*, *Kostermansia malayana Soegeng.*, and *Helicteres hirsuta Lour.*, all of which contained more than 160 long repeats (Fig. 6A; Table S6). The majority of long repeat were 30 to 45 bp in length, followed by those 45–60 bp long. Repeats of 60–75 bp and > 75 bp were relatively scarce or even absent in many species (Fig. 6B; Table S6).

### Codon usage pattern

**Fig. 7:**
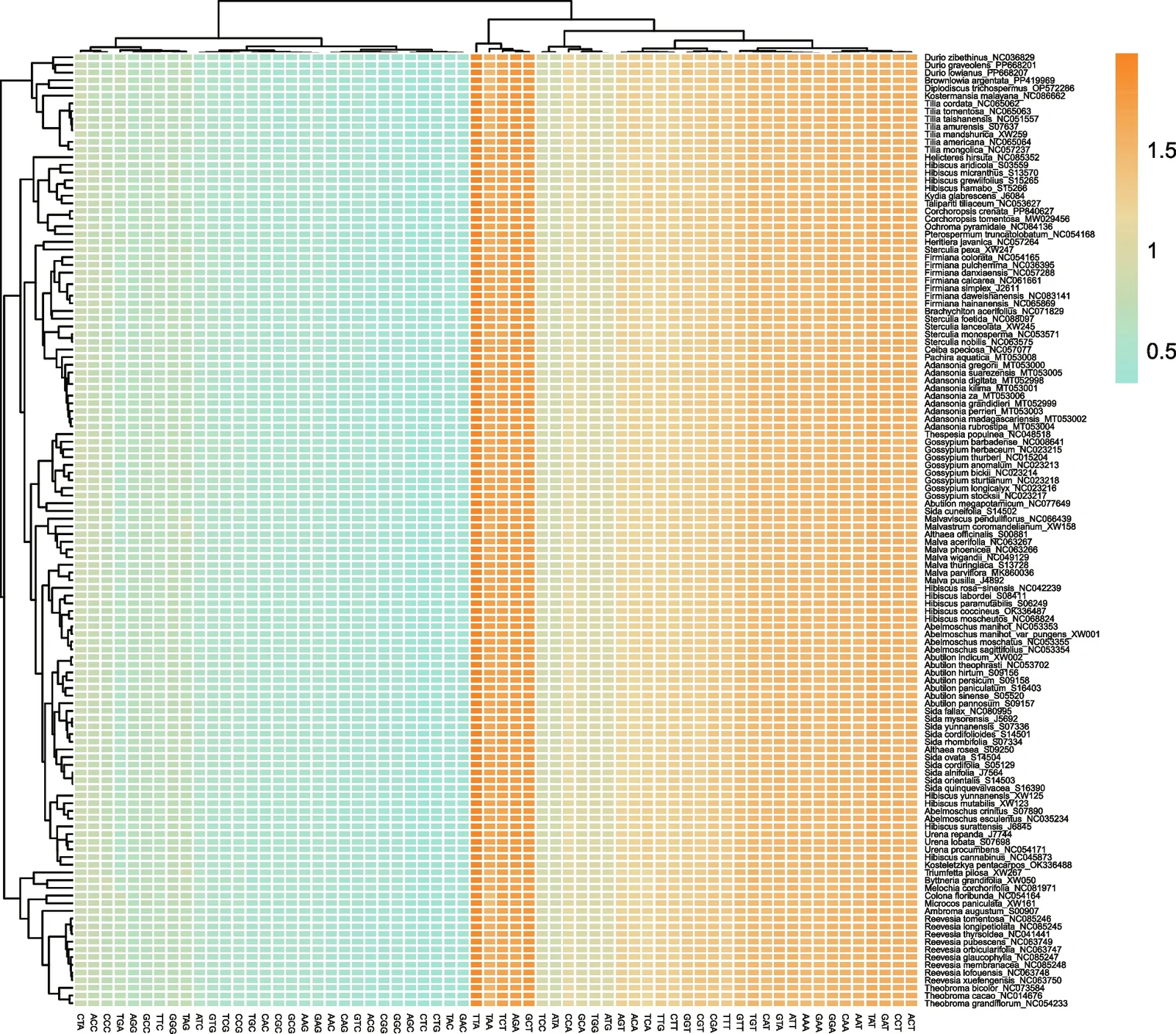
The value of relative synonymous codon usage (RSCU) for all protein-coding genes in 124 Malvaceae plastomes

The number of codons in protein-coding regions ranged from 18,603 to 21,419 across the plastomes of Malvaceae. The most frequently used codon was ATT (Ile) with usage ranging from 787 to 928. The highest relative synonymous codon usage (RSCU) value was for TTA (Leu; mean = 1.95). Of the 30 codons with RSCU values > 1.00, 29 ended with A/T, indicating a strong A/T ending preference. Only TGG (encoding tryptophan) and ATG (encoding methionine) showed RSCU = 1.00 across all species, indicating no codon usage bias (Fig. 7; Table S7).

### Selective pressure analysis

**Fig. 8:**
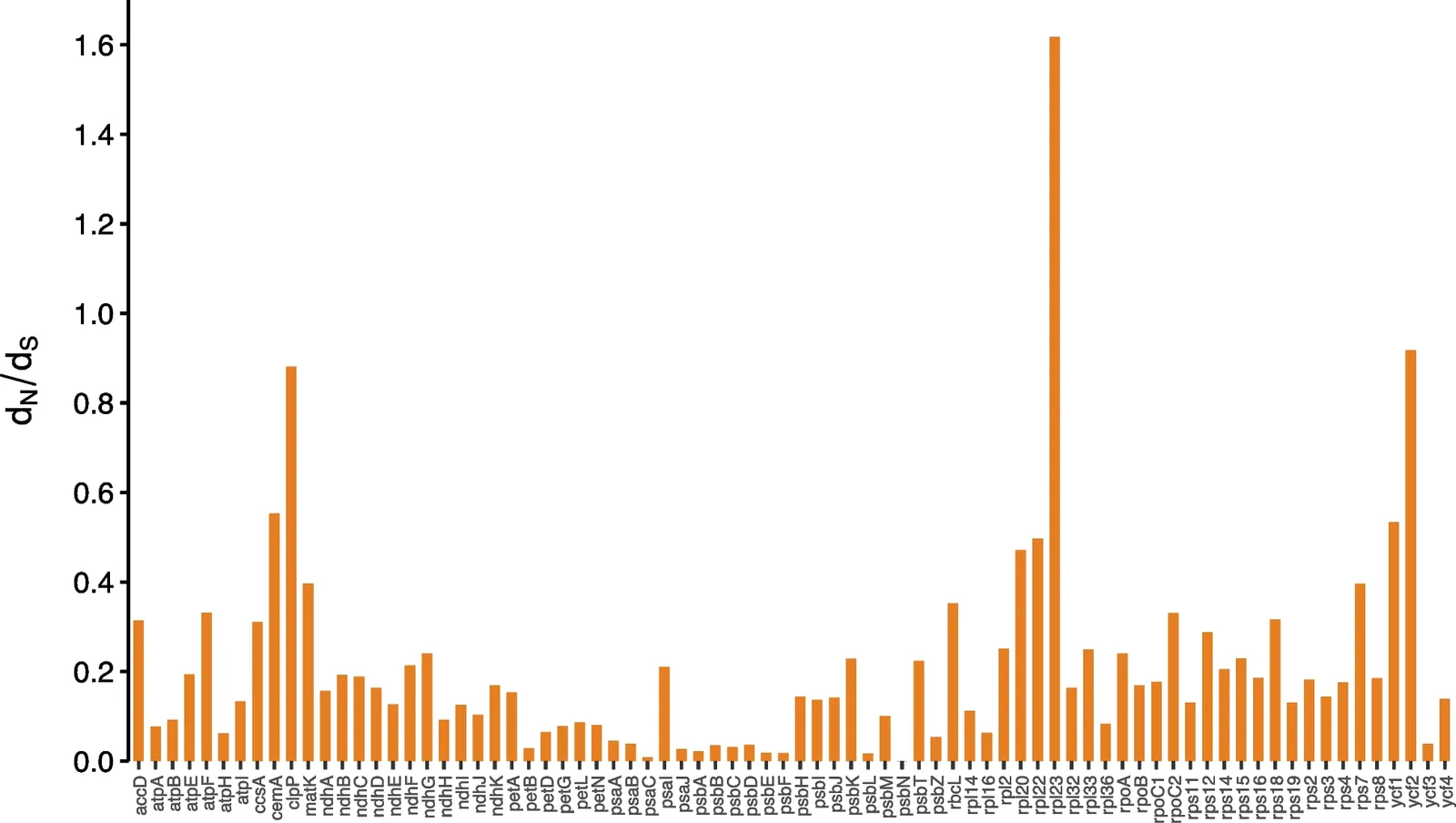
Non-synonymous/synonymous substitution rate ratios (dN/dS) for all protein-coding genes in 124 Malvaceae plastomes

The *dN/dS* ratios for 78 PCGs across 124 Malvaceae plastomes ranged from 0.0001 to 1.61737. *rpl23* showed *dN/dS* > 1.00, indicating positive selection, whereas *psbN* displayed a ratio near 0 (0.0001) (Fig. 8 and Table S8). Branch model analysis comparing M0 (one-ratio) and M2 (two-ratio) models indicated that most PCGs fit the M0 model better. In the Byttneriina clade, 12 genes (*accD*, *petD*, *psaI*, *rbcL*, *rpl14*, *rpl16*, *rpl22*, *rpl32*, *rpoB*, *rpoC1*, *rps18*, and *rps7*) fit M2 better, suggesting they underwent intensified purifying selection (Table S9). In the Malvadendrina clade, 13 genes (*accD*, *atpH*, *petD*, *petL*, *psaI*, *psbJ*, *rbcL*, *rpl14*, *rpl16*, *rpl22*, *rpl32*, *rpoB*, and *rpoC1*) also experienced intensified purifying selection, while four genes (*rps18*, *rps7*, *ycf1*, and *ycf3*) showed relaxed purifying selection. y*cf2* had an elevated *dN/dS* ratio (1.02823), suggesting accelerated evolution in this lineage (Table S10).

## Discussion

### Structural conservation and dynamic IR boundary evolution

The plastomes of Malvaceae exemplify the canonical, highly conserved quadripartite architecture typical of angiosperms, with gene content, order, and overall size (158,346–163,741 bp) conforming to patterns observed across land plants [49]. This structural stasis underscores strong functional constraints on core processes like photosynthesis and gene expression [49, 50]. However, beneath this overarching conservation lies a certain degree of microstructural variation, primarily driven by the dynamic expansion and contraction of the inverted repeat (IR) regions. Our analysis of 124 plastomes, representing an unprecedented taxon sampling across the family, systematically classifies these IR/SC boundary variations into five major types and 11 distinct subtypes, revealing a far more complex evolutionary landscape than previously documented for Malvaceae [19].

The predominant Type I configuration, found in 112 plastomes, confirms the general stability of IR boundaries in the family. Yet, the identification of five subtypes within Type I, particularly the novel Subtypes I-3 and I-5, provides compelling evidence for continuous and small-scale boundary fluctuations. These likely represent transitional states in the canonical "ebb and flow" model of IR evolution [51, 52], where minor shifts can precede larger structural changes. The discovery of lineage-specific Types (II-V) highlights independent evolutionary trajectories within Malvaceae. For instance, the significant IR expansion in *Abelmoschus* Medik. (Type II), which duplicates partial segments of *rpl16* and *ycf1*, contrasts sharply with the distinct IR contraction observed in *Durio* and *Kostermansia* (Type III), where *ndhF* and *ycf1* reside entirely within the SSC. The unique boundary configurations in *Pachira aquatica* (Type IV) and *Melochia corchorifolia* (Type V) further underscore that specific lineages have undergone accelerated plastome structural evolution. These boundary dynamics are not merely structural curiosities; they are a primary mechanism for plastome size variation and generate localized evolutionary hotspots. Genes spanning these junction sites, such as* ycf1* and *ndhF*, exhibit elevated nucleotide diversity (Pi), transforming them into valuable phylogenetic markers with resolving power exceeding that of standard conserved regions.

### Sequence divergence, repeat dynamics, and signatures of adaptive evolution

While plastomes are conserved, identifying regions of elevated divergence is crucial for developing effective molecular markers and understanding evolutionary drivers. Our study corroborates the universal pattern of higher sequence variability in non-coding regions than in coding regions [53], but significantly refines the catalog of hypervariable loci within Malvaceae. We identify six coding regions (e.g., *matK*, *ndhF*, and *ycf1*) and 14 non-coding spacers (e.g., *ndhF*-*rpl32*, *rpl32*-*trnL*, and *trnH*-*psbA*) as the most variable (Pi > 0.03 and > 0.05, respectively). Notably, we propose a tiered marker strategy: the protein-coding gene *ycf1* offers a stable, information-rich region for resolving deeper and intermediate-level relationships [54], while hypervariable non-coding spacers like *ndhF*-*rpl32* are ideal for fine-scale phylogenetics and species delimitation within recently diversified groups [55].

Analyses of repetitive elements revealed distinctive genomic signatures, particularly in the genus *Durio.* Although Malvaceae plastomes broadly follow angiosperm trends—featuring A/T-rich mononucleotide Simple Sequence Repeats (SSRs) and a pronounced A/T bias in codon usage [48, 56]—*Durio* species exhibited a remarkable proliferation of both SSRs and long repeats (forward and palindromic). This anomalous accumulation suggests either a lineage-specific relaxation of DNA repair mechanisms or a period of accelerated plastome evolution [57], providing a tangible genomic correlate for its distinct phylogenetic position. These repetitive elements likely act as recombination hotspots, contributing to the structural plasticity observed in *Durio* and serving as a rich source of lineage-specific molecular markers.

Selection pressure analyses across the family revealed that most protein-coding genes are under strong purifying selection, consistent with their essential functions. However, *rpl23* showed a *dN/dS* value greater than 1.00, suggesting a potential signal of positive selection or accelerated evolution in Malvaceae. As a component of the large ribosomal subunit encoded in the generally stable IR region, *rpl23* is typically highly conserved [58, 59]. Positive selection signals or elevated evolutionary rates of plastid *rpl23* have also been reported in other angiosperm lineages, such as Styracaceae and Lythraceae [60, 61], suggesting that accelerated evolution of this gene may have occurred independently in different plant groups. The elevated *dN/dS* value of *rpl23* in Malvaceae may reflect lineage-specific accelerated evolution of this gene, possibly associated with changes in the chloroplast translational apparatus. This pattern might be relevant to the ecological diversification of Malvaceae, including the colonization of arid habitats by some lineages such as Bombacoideae [62]. However, this hypothesis remains speculative and requires further testing with broader taxon sampling, nuclear genomic data, and functional evidence. Furthermore, we detected patterns of relaxed purifying selection on genes such as *rps18*, *rps7*, *ycf1*, and *ycf3* in specific clades (e.g., Malvadendrina). This relaxation may indicate a period of evolutionary plasticity, allowing for the accumulation of genetic variation that could serve as raw material for future adaptation, rather than representing a direct adaptive response itself.

### Phylogenomic insights and resolution of subfamilial relationships

Our phylogenomic analyses, based on the most densely sampled plastome dataset for Malvaceae to date, provide robust support for the family's backbone topology. The deep split into the Byttneriina (Byttnerioideae + Grewioideae) and Malvadendrina clades is unequivocally confirmed. Notably, for example, *Durio*, as a member of Helicteroideae, not only sits at the base of Malvadendrina but also possesses significantly more long repeats and SSRs in its plastome [63], suggesting that basal lineages may have experienced higher rates of DNA replication or recombination events. Within the Malvadendrina clade, our results strongly support Helicteroideae (represented by *Durio* and allies) as the earliest-diverging lineage (BS = 100% and PP = 1.00), a finding consistent with several plastome-based studies [16, 48] but contrasting with some inferences from nuclear data [15], which supported Dombeyoideae as the earliest-diverging lineage (BS = 100%) within the Malvadendrina clade. This cytonuclear discordance likely stems from complex evolutionary histories involving incomplete lineage sorting, hybridization, or introgression [64], highlighting the necessity of multi-genomic datasets for a complete phylogenetic understanding. This conflict cannot be attributed solely to technical or signal differences; it more likely reflects incomplete lineage sorting or ancient hybridization during the diversification of Malvaceae [17, 64]. This pattern is a manifestation of phylogenetic conflict that can be caused by plastid capture or nuclear introgression. Distinguishing these possibilities requires phylogenomic methods that can detect reticulate evolution and multi-locus analytical strategies. The deep divergence between Byttneriina and Malvadendrina coincides with warm phases from the late Cretaceous to Paleocene [14], and the basal position of Helicteroideae-whose extant members (e.g., *Durio*) often occupy tropical rainforests and exhibit distinctive fruit traits-suggests that early diversification within Malvaceae may have been linked to warm, humid environments and the rich ecological niches provided by such environments.

Our plastome phylogeny further clarifies other contentious relationships within the Malvadendrina clade. Sterculioideae is strongly supported as the next diverging lineage after Helicteroideae. Subsequently, Brownlowioideae is resolved as sister to a well-supported clade (BS = 99% and PP = 1.00) comprising Tilioideae and Dombeyoideae. This topology offers a clear resolution to previously inconsistent placements of these subfamilies [15, 65].

At the genus level, our data confirm the non-monophyly of both *Hibiscus* and *Sida* within Malvoideae, echoing long-standing taxonomic complexities [66–68]. The incongruent placement of *Theobroma* L. between our complete plastome and coding sequence (CDS) trees underscores a critical methodological insight: non-coding regions, which contribute significantly to the phylogenetic signal in complete plastome analyses, may be particularly important for resolving certain problematic nodes. Relying solely on CDS may result in a loss of resolution due to reduced information content. Therefore, the persistent non-monophyly and unresolved relationships within genera like *Hibiscus* and *Sida* point to likely recent radiations and/or pervasive hybridization. Furthermore, the non-monophyly of these genera suggests that morphological characters traditionally used to define them may be homoplastic, and that rapid species radiation in Malvoideae likely involved repeated convergent evolution of floral traits [69, 70]. challenges that will require integrated phylogenomic approaches using high-copy nuclear targets or whole-genome data to fully unravel.

## Conclusion

This study presents the most comprehensive plastomic analysis across Malvaceae to date, based on 128 plastomes (124 used for structural variation assessment). Our results revealed high conservation in genome size, structure, and gene content of Malvaceae plastomes. However, distinct evolutionary patterns were observed in *Durio*, which possessed significantly more long repeats and SSRs. Selection pressure analysis identified positive selection acting on the *rpl23* gene, suggesting its potential role in adaptive evolution to environmental stresses. Multiple hypervariable regions were identified as valuable resources for future phylogenetic and genetic marker development. Phylogenetic analysis strongly supports the monophyly of the nine subfamilies and resolved Helicteroideae as the first diverging lineage within the Malvadendrina clade. Nevertheless, our results revealed the non-monophyly of *Sida* and *Hibiscus*, and incongruences between datasets, indicating that relationships among some subfamilies and genera remain contentious. Future studies should use nuclear genomic data to further resolve these phylogenetic relationships.

## Supplementary Information

Supplementary Material 1.

Supplementary Material 2.

## Data Availability

The newly sequenced plastome sequences have been deposited in the CNGB Sequence Archive (CNSA) of the China National GeneBank DataBase (CNGBdb). The data are publicly available under project (CNP0008960) at https://db.cngb.org/data_resources/project/CNP0008960. Individual sample accession numbers range from CNS1464382 to CNS1464426, and the corresponding sequence accession numbers range from N_002032060 to N_002032104 (Supplemental Table S2).

## References

[1] Christenhusz MJM, Byng JW. The number of known plants species in the world and its annual increase. Phytotaxa. 2016;261:201–17.

[2] Ganesan SK, Middleton DJ, Wilkie P. A revision of *Pterospermum* (Malvaceae: Dombeyoideae) in Malesia. Edinburgh J Bot. 2020;77:161–241.

[3] Bayer C, Kubitzki K. Malvaceae. In: Kubitzki K, editor. The families and genera of vascular plants, vol. volume 5. Berlin: Springer; 2003. p. 225–311.

[4] Ekalu A, Habila JD. Phytochemistry, pharmacology and medicinal uses of *Cola* (Malvaceae) family: a review. Med Chem Res. 2020;29:2089–105.

[5] Li W, Chen X, Yu J, Zhu Y. Upgraded durian genome reveals the role of chromosome reshuffling during ancestral karyotype evolution, lignin biosynthesis regulation, and stress tolerance. Sci China Life Sci. 2024;67:1266–79. 10.1007/s11427-024-2580-3 38763999

[6] Bossa-Castro AM, Colli-Silva M, Pirani JR, et al. A phylogenetic framework to study desirable traits in the wild relatives of *Theobroma cacao* (Malvaceae). J Syst Evol. 2024;62:963–78.

[7] Dick CW, Bermingham E, Lemes MR, Gribel R. Extreme long-distance dispersal of the lowland tropical rainforest tree *Ceiba pentandra* L. (Malvaceae) in Africa and the Neotropics. Mol Ecol. 2007;16:3039–49. 10.1111/j.1365-294X.2007.03341.x 17614916

[8] Wang JH, Moore MJ, Wang H, Zhu ZX, Wang HF. Plastome evolution and phylogenetic relationships among Malvaceae subfamilies. Gene. 2021;765:145103. 10.1016/j.gene.2020.145103 32889057

[9] Alverson WS, Whitlock BA, Nyffeler R, Bayer C, Baum DA. Phylogeny of the core Malvales: evidence from *ndhF* sequence data. Am J Bot. 1999;86:1474–86. 10523287

[10] Bayer C, Fay MF, De Bruijn AY, Savolainen V, Morton CM, Kubitzki K, et al. Support for an expanded family concept of Malvaceae within a recircumscribed order Malvales: a combined analysis of plastid *atpB* and *rbcL* DNA sequences. Bot J Linn Soc. 1999;129(4):267–303.

[11] Angiosperm Phylogeny Group. An update of the Angiosperm Phylogeny Group classification for the orders and families of flowering plants: APG II. Bot J Linn Soc. 2003;141(4):399–436.

[12] Nyffeler R, Baum DA. Phylogenetic relationships of the durians (Bombacaceae-Durionieae or Malvaceae/Helicteroideae/Durionieae) based on chloroplast and nuclear ribosomal DNA sequences. Plant Syst Evol. 2000;224:55–82.

[13] Hernandez-Gutierrez R, Magallon S. The timing of Malvales evolution: incorporating its extensive fossil record to inform about lineage diversification. Mol Phylogenet Evol. 2019;140:106606. 10.1016/j.ympev.2019.106606 31472220

[14] Cvetković T, Areces-Berazain F, Hinsinger DD, Thomas DC, Wieringa JJ, Ganesan SK, et al. Phylogenomics resolves deep subfamilial relationships in Malvaceae s.l. G3 (Bethesda). 2021;11:jkab136. 10.1093/g3journal/jkab136 PMC8496235 33892500

[15] Conover JL, Karimi N, Stenz N, Ané C, Grover CE, Skema C, et al. A Malvaceae mystery: a mallow maelstrom of genome multiplications and maybe misleading methods? J Integr Plant Biol. 2019;61(1):12–31. 10.1111/jipb.12746 30474311

[16] Wu M, He L, Ma G, Zhang K, Yang H, Yang X. The complete chloroplast genome of *Diplodiscus trichospermus* and phylogenetic position of Brownlowioideae within Malvaceae. BMC Genomics. 2023;24:571. 10.1186/s12864-023-09680-z PMC10521492 37752438

[17] Colli-Silva M, Pérez-Escobar OA, Ferreira CDM, Costa MT, Gerace S, Coutinho TS, et al. Taxonomy in the light of incongruence: an updated classification of Malvales and Malvaceae based on phylogenomic data. Taxon. 2025;74:361–85.

[18] Geng LY, Jiang TY, Chen X, Li Q, Ma JH, Hou WX, et al. Plastome structure, phylogeny and evolution of plastid genes in *Reevesia* (Helicteroideae, Malvaceae). J Plant Res. 2024;137:589–604. 10.1007/s10265-024-01547-y 38739241

[19] Li J, Ye G, Liu H, Wang Z. Complete chloroplast genomes of three important species, *Abelmoschus moschatus*, *A. manihot* and *A. sagittifolius*: genome structures, mutational hotspots, comparative and phylogenetic analysis in Malvaceae. PLoS ONE. 2020;15:e0242591. 10.1371/journal.pone.0242591 PMC7688171 33237925

[20] Erixon P, Oxelman B. Whole-gene positive selection, elevated synonymous substitution rates, duplication, and indel evolution of the chloroplast *clpP1* gene. PLoS ONE. 2008;3:e1386. 10.1371/journal.pone.0001386 PMC2148103 18167545

[21] Cauz-Santos LA. Beyond conservation: the landscape of chloroplast genome rearrangements in angiosperms. New Phytol. 2025;247:2571–80. 10.1111/nph.70364 PMC12371153 40613318

[22] Palmer JD. Comparative organization of chloroplast genomes. Annu Rev Genet. 1985;19:325–54. 10.1146/annurev.ge.19.120185.001545 3936406

[23] Olmstead RG, Palmer JD. Chloroplast DNA systematics: a review of methods and data analysis. Am J Bot. 1994;81:1205–24.

[24] Jin JJ, Yu WB, Yang JB, Song Y, DePamphilis CW, Yi TS, et al. GetOrganelle: a fast and versatile toolkit for accurate de novo assembly of organelle genomes. Genome Biol. 2020;21:241. 10.1186/s13059-020-02154-5 PMC7488116 32912315

[25] Ruhlman TA, Jansen RK. The plastid genomes of flowering plants. In: Maliga P, editor. Chloroplast biotechnology: methods and protocols. Totowa, NJ: Humana Press; 2014. p. 3–38. 10.1007/978-1-62703-995-6_1 24599844

[26] Shen J, Zhang X, Landis JB, Zhang H, Deng T, Sun H, et al. Plastome evolution in *Dolomiaea* (Asteraceae, Cardueae) using phylogenomic and comparative analyses. Front Plant Sci. 2020;11:376. 10.3389/fpls.2020.00376 PMC7174903 32351518

[27] Xiong Y, Lei X, Xiong Y, Liu Y, Dong Z, Zhao J, et al. Factors contributing to organelle genomes size variation and the intracellular DNA transfer in Polygonaceae. BMC Genomics. 2024;25:994. 10.1186/s12864-024-10914-x PMC11515532 39443865

[28] Li J, Lu SS, Bi Y, Jiang YM, Feng LD, He J. Assembly and analysis of the mitochondrial genome of *Hippophae rhamnoides* subsp. *sinensis*, an important ecological and economic forest tree species in China. Plants (Basel). 2025;14:2170. 10.3390/plants14142170 PMC12299680 40733405

[29] Zhou H, Huang X, Liu J, Ding J, Xu K, Zhu W, et al. De novo phased genome assembly, annotation and population genotyping of *Alectoris chukar*. Sci Data. 2024;11:162. 10.1038/s41597-024-02991-0 PMC10837146 38307880

[30] Doyle JJ, Doyle JLA. rapid DNA isolation procedure for small quantities of fresh leaf tissue. Phytochem Bull. 1987;19:11–5.

[31] Qu XJ, Moore MJ, Li DZ, Yi TS. PGA: a software package for rapid, accurate, and flexible batch annotation of plastomes. Plant Methods. 2019;15:50. 10.1186/s13007-019-0435-7 PMC6528300 31139240

[32] Zhang NN, Stull GW, Zhang XJ, Fan SJ, Yi TS, Qu XJ. PlastidHub: an integrated analysis platform for plastid phylogenomics and comparative genomics. Plant Divers. 2025;47:544–60. 10.1016/j.pld.2025.05.005 PMC12302489 40734827

[33] Tillich M, Lehwark P, Pellizzer T, Ulbricht-Jones ES, Fischer A, Bock R, et al. GeSeq—versatile and accurate annotation of organelle genomes. Nucleic Acids Res. 2017;45:W6–11. 10.1093/nar/gkx391 PMC5570176 28486635

[34] Kearse M, Moir R, Wilson A, Stones-Havas S, Cheung M, Sturrock S, et al. Geneious basic: an integrated and extendable desktop software platform for the organization and analysis of sequence data. Bioinformatics. 2012;28:1647–9. 10.1093/bioinformatics/bts199 PMC3371832 22543367

[35] Greiner S, Lehwark P, Bock R. OrganellarGenomeDRAW (OGDRAW) version 1.3. 1: expanded toolkit for the graphical visualization of organellar genomes. Nucleic Acids Res. 2019;47:W59–64. 10.1093/nar/gkz238 PMC6602502 30949694

[36] Darling ACE, Mau B, Blattner FR, Perna NT. Mauve: multiple alignment of conserved genomic sequence with rearrangements. Genome Res. 2004;14:1394–403. 10.1101/gr.2289704 PMC442156 15231754

[37] Li H, Guo Q, Xu L, Gao H, Liu L, Zhou X. CPJSdraw: analysis and visualization of junction sites of chloroplast genomes. PeerJ. 2023;11:e15326. 10.7717/peerj.15326 PMC10182761 37193025

[38] Dubchak I, Ryaboy DV. VISTA family of computational tools for comparative analysis of DNA sequences and whole genomes. In: Gene mapping, discovery, and expression: methods and protocols. Totowa, NJ: Humana Press; 2006. p. 69–89. 10.1385/1-59745-097-9:69 16888351

[39] Huang L, Yu H, Wang Z, Xu W. CPStools: a package for analyzing chloroplast genome sequences. iMetaOmics. 2024;1:e25. 10.1002/imo2.25 PMC12806284 41676121

[40] Kurtz S, Schleiermacher C. REPuter: fast computation of maximal repeats in complete genomes. Bioinformatics. 1999;15:426–7. 10.1093/bioinformatics/15.5.426 10366664

[41] Kurtz S, Choudhuri JV, Ohlebusch E, Schleiermacher C, Stoye J, Giegerich R. REPuter: the manifold applications of repeat analysis on a genomic scale. Nucleic Acids Res. 2001;29:4633–42. 10.1093/nar/29.22.4633 PMC92531 11713313

[42] Zhang D, Gao F, Jakovlić I, Zou H, Zhang J, Li WX, et al. PhyloSuite: an integrated and scalable desktop platform for streamlined molecular sequence data management and evolutionary phylogenetics studies. Mol Ecol Resour. 2020;20:348–55. 10.1111/1755-0998.13096 31599058

[43] Stamatakis A. RAxML version 8: a tool for phylogenetic analysis and post-analysis of large phylogenies. Bioinformatics. 2014;30:1312–3. 10.1093/bioinformatics/btu033 PMC3998144 24451623

[44] Ronquist F, Teslenko M, Van Der Mark P, Ayres DL, Darling A, Höhna S, et al. MrBayes 3.2: efficient Bayesian phylogenetic inference and model choice across a large model space. Syst Biol. 2012;61:539–42. 10.1093/sysbio/sys029 PMC3329765 22357727

[45] Yang Z. PAML 4: phylogenetic analysis by maximum likelihood. Mol Biol Evol. 2007;24:1586–91. 10.1093/molbev/msm088 17483113

[46] Yang Z, Wong WSW, Nielsen R. Bayes empirical Bayes inference of amino acid sites under positive selection. Mol Biol Evol. 2005;22:1107–18. 10.1093/molbev/msi097 15689528

[47] Gao F, Chen C, Arab DA, Du Z, He Y, Ho SY. EasyCodeML: a visual tool for analysis of selection using CodeML. Ecol Evol. 2019;9:3891–8. 10.1002/ece3.5015 PMC6467853 31015974

[48] Zhong Y, Bai B, Sun Y, Wen K, Qiao Y, Guo L, et al. Comparative genomics and phylogenetic analysis of six Malvaceae species based on chloroplast genomes. BMC Plant Biol. 2024;24:1245. 10.1186/s12870-024-05974-w PMC11670485 39722018

[49] Daniell H, Lin CS, Yu M, Chang WJ. Chloroplast genomes: diversity, evolution, and applications in genetic engineering. Genome Biol. 2016;17:134. 10.1186/s13059-016-1004-2 PMC4918201 27339192

[50] Wicke S, Schneeweiss GM, Depamphilis CW, Müller KF, Quandt D. The evolution of the plastid chromosome in land plants: gene content, gene order, gene function. Plant Mol Biol. 2011;76:273–97. 10.1007/s11103-011-9762-4 PMC3104136 21424877

[51] Goulding SE, Wolfe KH, Olmstead RG, Morden CW. Ebb and flow of the chloroplast inverted repeat. Mol Gen Genet. 1996;252:195–206. 10.1007/BF02173220 8804393

[52] Fu N, Ji M, Rouard M, Yan HF, Ge XJ. Comparative plastome analysis of Musaceae and new insights into phylogenetic relationships. BMC Genomics. 2022;23:223. 10.1186/s12864-022-08454-3 PMC8939231 35313810

[53] Shaw J, Lickey EB, Beck JT, Farmer SB, Liu W, Miller J, et al. The tortoise and the hare II: relative utility of 21 noncoding chloroplast DNA sequences for phylogenetic analysis. Am J Bot. 2005;92:142–66. 10.3732/ajb.92.1.142 21652394

[54] Dong W, Xu C, Li C, Sun J, Zuo Y, Shi S, et al. Ycf1, the most promising plastid DNA barcode of land plants. Sci Rep. 2015;5:8348. 10.1038/srep08348 PMC4325322 25672218

[55] Shaw J, Shafer HL, Leonard OR, Kovach MJ, Schorr M, Morris AB. Chloroplast DNA sequence utility for the lowest phylogenetic and phylogeographic inferences in angiosperms: the tortoise and the hare IV. Am J Bot. 2014;101:1987–2004. 10.3732/ajb.1400398 25366863

[56] Wang Y, Jiang D, Guo K, Zhao L, Meng F, Xiao J, et al. Comparative analysis of codon usage patterns in chloroplast genomes of ten *Epimedium* species. BMC Genom Data. 2023;24:3. 10.1186/s12863-023-01104-x PMC9830715 36624369

[57] Zhang J, Ruhlman TA, Sabir JS, Blazier JC, Weng ML, Park S, et al. Coevolution between nuclear-encoded DNA replication, recombination, and repair genes and plastid genome complexity. Genome Biol Evol. 2016;8:622–34. 10.1093/gbe/evw033 PMC4824065 26893456

[58] Wen J, Wu BC, Li HM, Zhou W, Song CF. Plastome structure and phylogenetic relationships of genus *Hydrocotyle* (Apiales): provide insights into the plastome evolution of *Hydrocotyle*. BMC Plant Biol. 2024;24:778. 10.1186/s12870-024-05483-w PMC11325595 39148054

[59] Xu P, Meng M, Wu F, Zhang J. A comparative plastome approach enhances the assessment of genetic variation in the *Melilotus* genus. BMC Genom. 2024;25:556. 10.1186/s12864-024-10476-y PMC11149310 38831327

[60] Cai XL, Landis JB, Wang HX, Wang JH, Zhu ZX, Wang HF. Plastome structure and phylogenetic relationships of Styracaceae (Ericales). BMC Ecol Evol. 2021;21:103. 10.1186/s12862-021-01827-4 PMC8161964 34049486

[61] Gu C, Ma L, Wu Z, Chen K, Wang Y. Comparative analyses of chloroplast genomes from 22 Lythraceae species: inferences for phylogenetic relationships and genome evolution within Myrtales. BMC Plant Biol. 2019;19:281. 10.1186/s12870-019-1870-3 PMC6595698 31242865

[62] Zizka A, Carvalho-Sobrinho JG, Pennington RT, Queiroz LP, Alcantara S, Baum DA, et al. Transitions between biomes are common and directional in Bombacoideae (Malvaceae). J Biogeogr. 2020;47:1310–21.

[63] Cheon SH, Jo S, Kim HW, Kim YK, Sohn JY, Kim KJ. The complete plastome sequence of *durian*, *Durio zibethinus* L. (Malvaceae). Mitochondrial DNA B. 2017;2:763–4. 10.1080/23802359.2017.1398615 PMC7800296 33490476

[64] Hernández-Gutiérrez R, van den Berg C, Granados Mendoza C, Peñafiel Cevallos M, Freire ME, Lemmon EM, et al. Localized phylogenetic discordance among nuclear loci due to incomplete lineage sorting and introgression in the family of cotton and cacao (Malvaceae). Front Plant Sci. 2022;13:850521. 10.3389/fpls.2022.850521 PMC9043901 35498660

[65] Hernández-Gutiérrez R, Mendoza CG, Magallón S. Low-copy nuclear genes reveal new evidence of incongruence in relationships within Malvaceae sl. Syst Bot. 2021;46:1042–52.

[66] Pejhanmehr M. Phylogenetic affinities among *Sida* species and allied genera (Malvaceae: Malveae), and examination of *Sida fallax* within the hawaiian islands and throughout the pacific [PhD thesis]. Honolulu: University of Hawai’i at Mãnoa; 2022.

[67] Hanes MM, Blanchard OJ, Valencia DJ, McLay T, Abbott JR, McDaniel SF, et al. Phylogenetic relationships within tribe Hibisceae (Malvaceae) reveal complex patterns of polyphyly in *Hibiscus* and *Pavonia*. Syst Bot. 2024;49:77–116.

[68] Lei HL, Li RZ, Cai J, Yang JB, Zhang ZR, Li DZ, et al. Plastomes evolution and phylogenetic analysis of the Malvatheca clade (Malvaceae). Guihaia. 2025;45:2241–54.

[69] Khalik KA, Al-Ruzayza S, Farid A. Taxonomic significances of seed morphology in some tribes of subfamily Malvoideae (Malvaceae) in Saudi Arabia. Aust J Crop Sci. 2021;15:1204–16.

[70] Ruan CJ, Chen SC, Li Q, Teixeira da Silva JA. Adaptive evolution of context-dependent style curvature in some species of the Malvaceae: a molecular phylogenetic approach. Plant Syst Evol. 2011;297:57–74.

