# Supplementary material for: Plastome evolution and phylogenomic insights from representative taxa across all nine subfamilies of Malvaceae
Source: BMC Plant Biol. 2026 Jul 18;26:1765. doi: 10.1186/s12870-026-09562-y (PMC13663193; doi:10.1186/s12870-026-09562-y)
Supplement: Supplementary file 1 [file 12870_2026_9562_MOESM1_ESM.pdf]

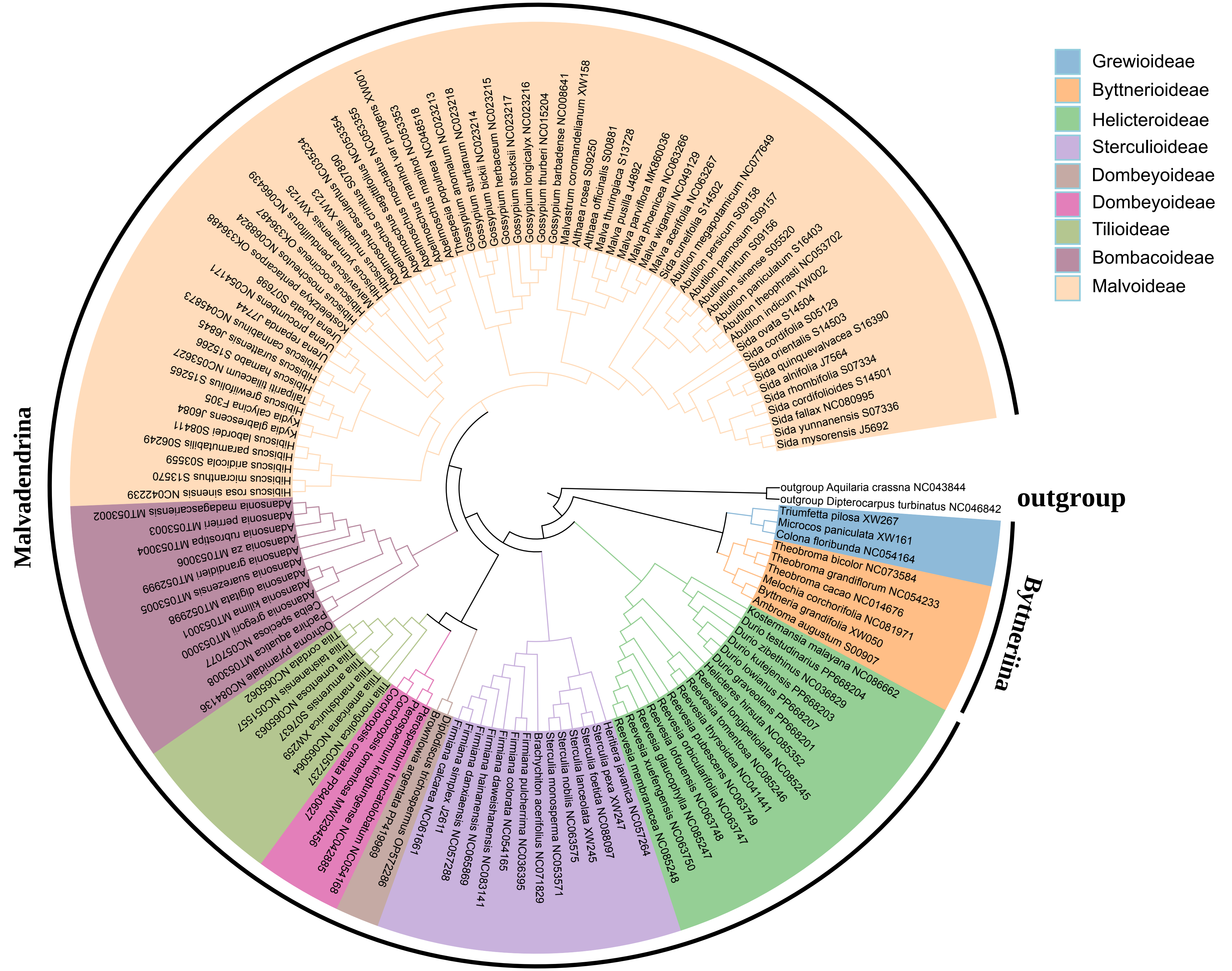

Fig S2 Bayesian inference phylogeny of Malvaceae. inferred from complete plastome sequences of 130 taxa, including 128 Malvaceae samples representing 127 species and two outgroups. Posterior probability values are shown at nodes; nodes without indicated values received a posterior probability of 1.00.

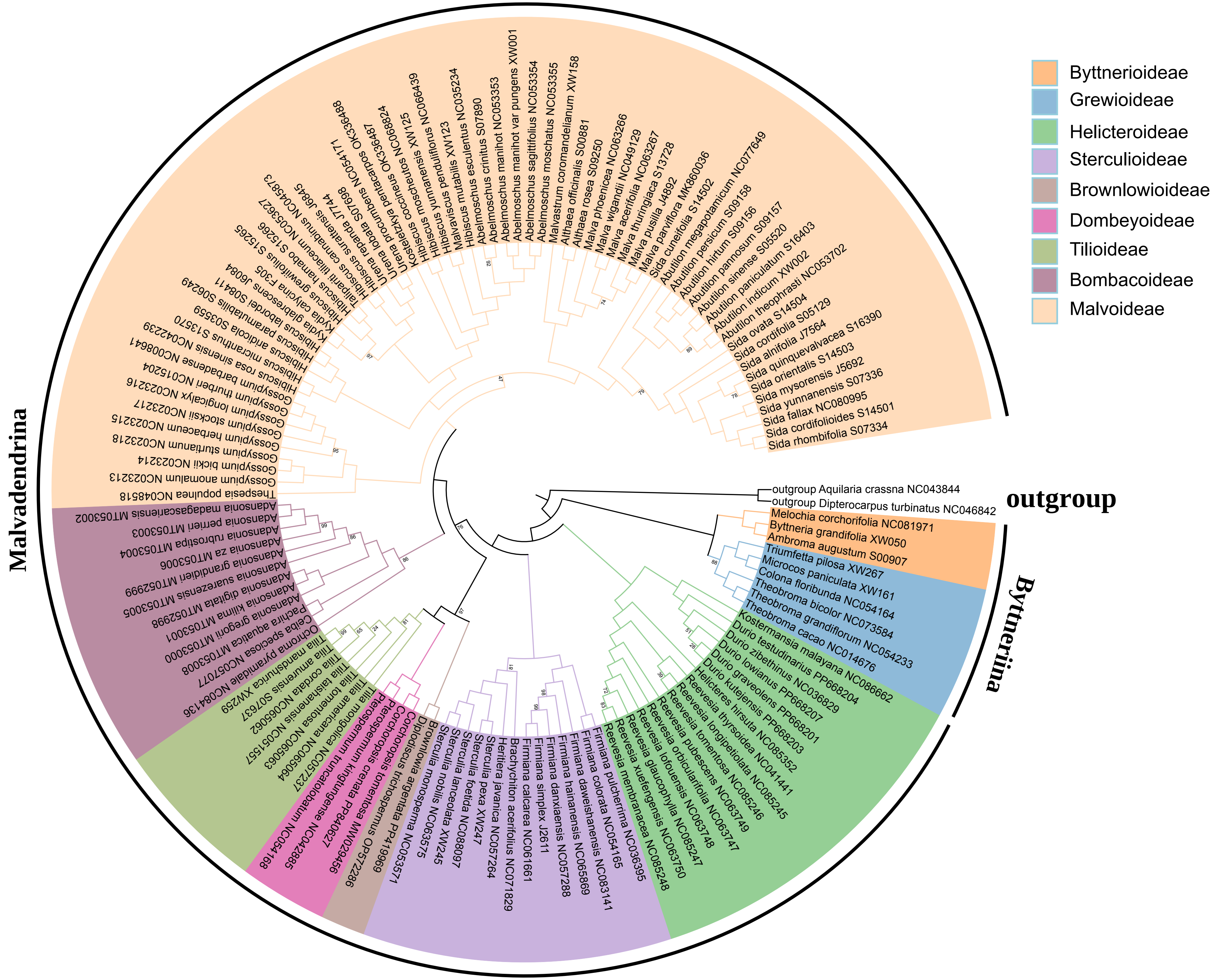

Fig S3 Maximum likelihood phylogeny of Malvaceae. inferred from the protein-coding sequence (CDS) matrix of 130 taxa, including 128 Malvaceae samples representing 127 species and two outgroups. Bootstrap support values are shown at nodes; nodes without indicated values received 100% support.

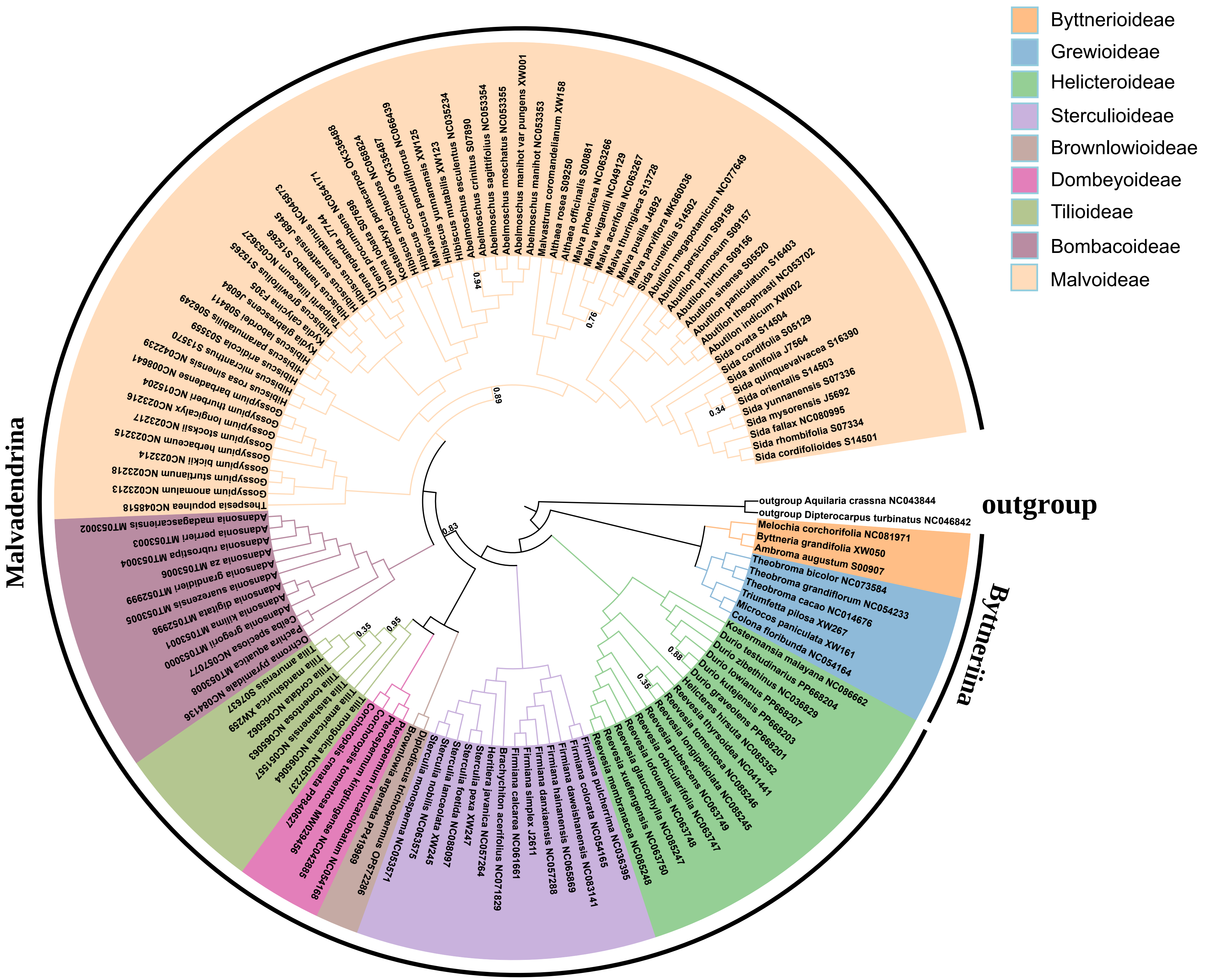

Fig S4 Bayesian inference phylogeny of Malvaceae. inferred from the protein-coding sequence (CDS) matrix of 130 taxa, including 128 Malvaceae samples representing 127 species and two outgroups. Posterior probability values are shown at nodes; nodes without indicated values received a posterior probability of 1.00.

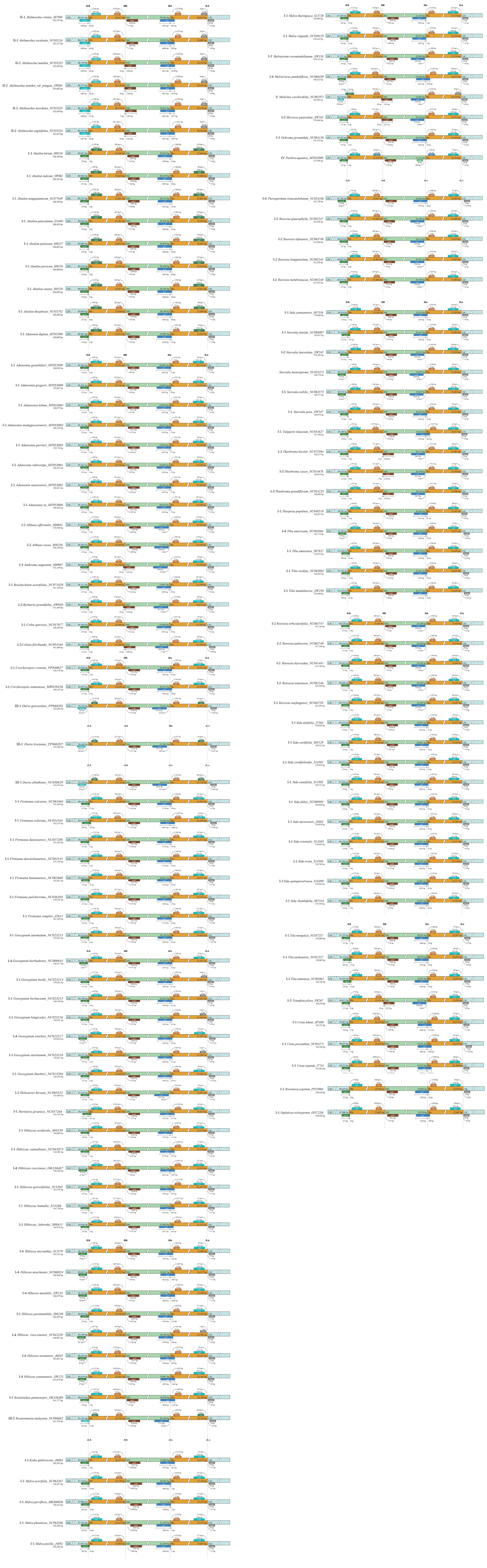

Fig S5 Comparative Analysis of IR Boundaries Across all 124 Malvaceae Samples.

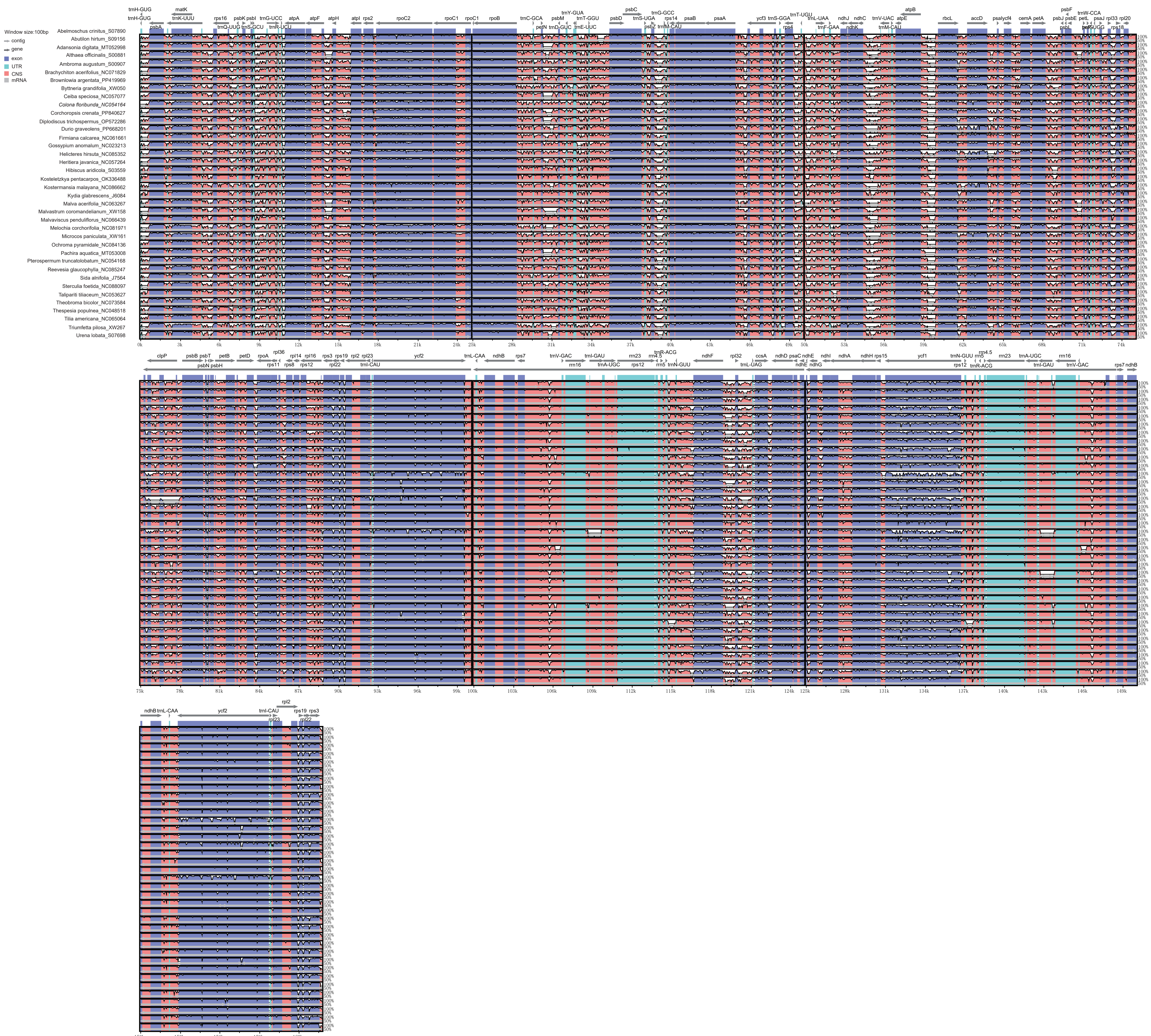

Fig S6 Complete plastid genomes of representative species from 38 Malvaceae genera were aligned and analyzed using mVISTA.
